# Perceived oral health interventions by medical providers in Gugulethu, South Africa

**DOI:** 10.1371/journal.pone.0233437

**Published:** 2020-05-26

**Authors:** R. Frederick Lambert, Amy Yu, Catherine Orrell, Jessica E. Haberer

**Affiliations:** 1 Boston Children’s Hospital, Boston, Massachusetts, United States of America; 2 Harvard School of Dental Medicine, Harvard University, Boston, Massachusetts, United States of America; 3 Desmond Tutu HIV Foundation, Institute of Infectious Disease and Molecular Medicine, University of Cape Town, Cape Town, South Africa; 4 Center for Global Health, Massachusetts General Hospital, Boston, Massachusetts, United States of America; 5 Harvard Medical School, Harvard University, Boston, Massachusetts, United States of America; Institute of Mental Health, SINGAPORE

## Abstract

**Introduction:**

The purpose of this study was to explore factors that impact patients’ ability to access high quality, expeditious oral health care by understanding medical professionals’ knowledge of oral health, the care they provide to patients presenting with oral health complaints, and their perceptions of potential interventions to improve oral health care delivery.

**Methods:**

We conducted in depth qualitative interviews, which were analyzed using an inductive content analytical approach. The study was conducted in Gugulethu, a community located outside of Cape Town, South Africa. Local public sector health services provided free-of-charge are the main source of primary health and dental care for this population. Participants included the following medical providers: doctors, clinical nurse practitioners, professional nurses, and health promoters.

**Results:**

Identified themes fell within the three broad subject areas: oral health knowledge, patient care, and potential interventions. Themes within oral health knowledge included (1) personal responsibility for hygiene, (2) routine oral health care, (3) lack of knowledge among medical professionals, (4) poverty, and (5) an oral-systemic connection. Participants cited both ‘clinical care knowledge’ and/or ‘uncertainty’ about patient care for oral health complaints. Participants independently suggested interventions in three broad areas: (1) education, (2) expanded provider roles, and (3) colocation of services.

**Conclusions:**

Our findings suggest that a variety of interventions, ranging from high to low resource investment, may impact access to and utilization of oral health services and thereby result in improved patient care. Future studies should develop and evaluate the suggested interventions in a range of care settings.

## Introduction

The World Health Organization (WHO) recognizes the need to address the epidemic of poor oral health, acknowledging poverty, inequality, and systemic disease as contributing factors, in addition to traditional oral health risk factors such as smoking and diet [1]. Currently an estimated 3.5 billon people live with oral health conditions ranging from caries to severe periodontal disease and even edentulism [2]. In 2015, over 2 billion people worldwide (approximately 35% of the population) were living with untreated dental caries on permanent teeth, making caries one of the most common health conditions [3]. Moreover, severe periodontal disease affects around 10% of the global population and rates have remained stable since 1990, making it one of the top ten most common health conditions [3]. Poor oral health can also contribute to systemic diseases, such as cardiovascular disease and diabetes, and may be debilitating to both individuals and communities [4]. Yet, oral diseases are preventable or easily treated early in the disease process. Current health care delivery models, however, often constrain early diagnosis and treatment due to a limited number of available providers and treatment options and/or a lack of understanding of available services [5–7].

Access to high quality oral health care is of particular concern in low-income countries and resource-poor communities due to limited infrastructure and a shortage trained oral health providers serving the public sector. The South African health care system, for example, faces ongoing challenges with access to and utilization of care in the public sector, and high-quality oral health services are no exception [8–11]. Previous work by our group [12] and others [13] examined patient experiences accessing oral health care in township settings (resource-poor communities of low socioeconomic status) surrounding Cape Town; we found that common knowledge of care delivery among patients consisted almost entirely of tooth extractions, which dissuaded community members from seeking oral health care [12,13] Additionally, our study found that patients with oral health complaints may present late in disease progression to health centers that lack oral health services. Lack of patient knowledge of oral health care services, as well as lack of access to and availability of preventive care, contributed to this finding. Patients presenting to these facilities were often provided with suboptimal treatment or referrals to oral health clinics where limited treatments are available or were turned away. New models for improved dental care and outcomes in resource-poor settings are needed.

To understand the key elements for designing such models, we conducted a qualitative study among medical providers in a Cape Town township. We explored factors that impact patients’ ability to access high quality and expeditious oral health care by understanding medical professionals’ knowledge of oral health, the care they provide to patients presenting with oral health complaints, and their perceptions of potential interventions to improve oral health care delivery.

## Methods

### Study site

Qualitative interviews took place in January 2019 in Gugulethu, a township community located outside of Cape Town, South Africa. Gugulethu has a population of approximately 100,000, among whom socioeconomic status is generally low. Local public sector health services are provided free-of-charge and are the main source of primary health and oral health care for this population, as well as those residing in the surrounding townships such as Crossroads, Nyanga, and Khayelitsha [14]. Residents of Gugulethu may seek medical services in neighboring townships as well. The study recruited from the Gugulethu Community Health Centre (GCHC), which is a primary health care facility broadly representative of the spectrum of primary care services across similar townships surrounding Cape Town. In Gugulethu, free oral health services are only provided at once facility called NY1 Clinic, which is affiliated with GCHC but located approximately one kilometer away.

### Sampling and recruitment

The study population consisted of health care providers currently working at GCHC. Eligible participants included doctors, clinical nurse practitioners, professional nurses, and health promoters (i.e., lay individuals who provide education on relevant health care topics to patients in the waiting room). All providers who had practiced at GCHC for greater than one month were interviewed.

### Qualitative data collection

Prior to any data collection, the interviewer (RFL) established rapport and trustworthiness at the clinic through several informal visits. His credibility was supported through an introduction by author CO, who has worked with the community for 20 years. To prevent reflexivity and bracketing, the RFL reassured staff that their opinions were highly valued and should not be influenced by himself or others. A single, one hour long, in-depth, semi-structured, qualitative interview was conducted with each participant at the time of enrollment. Open-ended questions elicited (1) knowledge of oral health, (2) experience with caring for patients with oral health conditions, and (3) perceptions of interventions aiming to increase access to and utilization of oral health care. A R100.00 (approximately US $7.00) shopping voucher was issued to each participant in appreciation of his or her time. Interviews were conducted in a private location in English (English is routinely used among health care professions and all participants were fluent in it) and were audio-recorded with the participant’s permission.

### Data analysis

Factors influencing access to oral health care were identified using an inductive conventional content analytical approach based on grounded theory [15]. Using NVivo 12.2.0, two investigators (RFL, AY) thematically coded the first five interviews and a codebook was created. This codebook was iteratively updated to represent new, recurrent themes, pulled directly from the text data, which were then applied to all transcripts. The influence, relevance, and meaning of each theme were discussed among authors RFL, JEH, and AY and were then grouped into descriptive categories. The participant’s thoughts and beliefs that comprise each theme were highlighted by specifically chosen quotations from the interviews. Full transcripts of the interviews and the codebook are available upon request and will be redacted appropriately to maintain anonymity of participants.

### Ethical approval

This study was approved by the University of Cape Town Faculty of Health Sciences Human Research Ethics Committee, Cape Town, South Africa (HREC REF: 643/2018), the Western Cape Provincial Health Research Committee (Reference: WC_201510_035), Partners Healthcare Institutional Review Board, Boston, MA (Protocol#: 2018P002269), and Harvard T.H. Chan School of Public Health Office of Human Research Administration, Boston, MA (Protocol#: IRB18-2098). Written consent was obtained from all participants.

## Results

### Participant characteristics

A total of 20 participants were invited to enroll and all agreed to the interview (Table 1). Thirty percent of the participants interviewed were doctors, 40% clinical nurse practitioners, 25% professional nurses, and 5% health promoters. Seventy-five percent of the participants were female. Participants reported generally seeing an average of 29 patients per day and had spent an average of 2.7 years (standard deviation: 2.1 years) working as health care professionals at GCHC.

**Table 1:** Participant characteristics.

Demographics	Men	Women	Total
Total	5	15	20
Doctor	3	3	6
Clinical Nurse Practitioner	2	6	8
Professional Nurse	0	5	5
Health Promoter	0	1	1
Average length of employment in years	1.9 (SD = 0.9)	3.0 (SD = 2.4)	2.7 (SD = 2.1)
Average number of patients treated per day	30 (SD = 3.5)	28 (SD = 7)	29 (SD = 6)

SD = standard deviation.

### Qualitative interview results

Thematic saturation was achieved with the interviewed sample; multiple themes were identified within the three broad subject areas: oral health knowledge, patient care for oral health complaints, and potential interventions. First, within content area of oral health knowledge, the following themes were identified: (1) personal responsibility for hygiene, (2) need for routine dental/oral heath care, (3) lack of knowledge among medical professionals, (4) impact of poverty on oral health, and (5) an oral-systemic connection. Discussions of patient care elicited themes falling under concepts of ‘clinical care knowledge’ or ‘uncertainty in managing patients with oral health complaints.’ The concept of ‘clinical care knowledge’ contained the sub-themes (1) chronic disease management, (2) oral health counseling, and (3) limited access and resources. ‘Uncertainty in managing patients with oral health complaints’ included (1) insufficient training, (2) referral and available services, and (3) follow-up. Finally, participants independently suggested interventions in three broad areas: (1) education, (2) expanded provider roles, and (3) colocation of services.

### Oral health knowledge

#### Personal responsibility for hygiene

Participants generally described oral heath as dependent on patients taking responsibility for their own care. Each participant stressed in importance of oral hygiene, including brushing and flossing in preventing oral disease. Participants stressed that oral hygiene was an important part of home care:

“Yes, so for me, it would be like I think of things that we do at home, brushing our teeth, flossing regularly.” (Doctor)

few participants mentioned the impact of diet on oral health. These participants noted that foods rich in sugar and acid contribute to the progression of caries and, therefore, patients should minimize consumption of sweets and related foods.

“The fizzy drinks, drinks like Coke, those that have acids, all those contribute in causing the teeth to decay. Then sweet things like cakes and sweets, and then when you chew the sweets every time, they cause those things.” (Professional nurse)

#### Routine dental/oral health care

Participants described annual or biannual exams and cleanings with a dentist as an integral aspect of good oral health. They acknowledged limited access to such services among their patients or uncertainty as to whether members of the Gugulethu community have access to or value annual oral exams.

“The mouth must be clean, and there must be fresh breath, not smelly breath, and then you must wash your teeth. It’s advised to go to the dentist six monthly, of which it’s not happening.” (Clinical nurse practitioner)

“They are not going because if there is no pain, if there is no trouble with their teeth, they tend to let it go. It’s ignorance, or because those services are full, so if you always go there, you are always turned away. They say it’s full today, come tomorrow, and then you tend to say hey, anyway, my teeth are not sore. I can just stay with my teeth as they are, until I have got a problem.” (Clinical nurse practitioner)

Participants understood the importance of regular dental visits and cleanings and stated that these exams are central to disease prevention.

“Routine or a regular follow up with a dentist or a dental hygienist, just to check up on things and sort of almost manage things prophylactically before they would become an issue, yes.” (Doctor)

#### Lack of knowledge among medical professionals

Participants explained that they do not have solid foundational knowledge of oral health. This lack of knowledge varied and related to all oral health, prevention of oral disease, and/or diagnosis and treatment of conditions affecting the oral cavity. One participant explained how the poor oral health status of patients underscores their own lack of oral health knowledge.

“…when I see patients with obviously very poor dental hygiene, I do sometimes question my training a little bit. Why, as a doctor, don’t I know more about the teeth and the conditions of the mouth and gums and teeth, in specific.” (Doctor)

#### An oral-systemic health connection

Although participants expressed uncertainty regarding their knowledge of oral health, they were able to explain the relationship between systemic and oral health. They commented that systemic diseases, both infectious (*e*.*g*., HIV) and noncommunicable (*e*.*g*., diabetes), can manifest in the oral cavity or how oral disease can impact overall health. One participant suggested that prevention of oral disease may help prevent systemic disease.

“I have heard that there are definitely risks with poor oral hygiene, and there are risks associated with heart disease and things. I mean, essentially, if we are trying to prevent these things, the more routes you can try to prevent heart disease, the better. It’s not rocket science, like brushing your teeth and looking after your oral health.” (Doctor)

#### Understanding the impact of poverty on oral health

Participants suggested that poverty impacts their patients in countless ways, including oral health. Some stated that poverty directly contributes to poor oral health status. One participant questioned whether patients have access to public resources, such as water and sanitation, which may make oral hygiene difficult.

“…do people have access to like water and sanitation, and are they able to access basic things like toothbrushes and toothpaste, and are they able to brush their teeth and look after their oral health or dental health?” (Doctor)

“I said you must clean your mouth so many times, and maybe they don’t have the resources to do so. If I don’t work, and I have this toothpaste, I would want to use it once a day rather than using it twice a day. If I stay in the bundus [rural areas], there is no water. I wouldn’t want to use that water to rinse my mouth each and every time I have eaten. I would rather save the water and only wash my mouth in the morning.” (Professional Nurse)

Another participant suggested that patients do not have the economic resources to afford supplies required for basic oral hygiene.

“I would say maybe it’s just a social economic status, not being able to afford toothpaste and stuff, and other oral cleaning agents” (Clinical nurse practitioner)

While participants stressed personal responsibility for hygiene and understood the importance of the oral-systemic connection, they were also aware of the barriers that their patients faced contributing to poor oral health.

### Addressing patient care for oral health complaints

#### Clinical care knowledge

In addition to the aspects of oral health knowledge noted above, participants expressed knowledge regarding care delivery for oral health. Many of the cases managed by the participants of this study involved chronic disease. Chronic care does exist but does not currently include oral health. Participants discussed treatment of patients with chronic illnesses, such as diabetes and hypertension, using strategies including lifestyle modification and dietary changes.

“The diet, you must eat less sweets and less fat. What you have at home, you must not always fry. You must always cook it, and we must always have some veggies on our plates. We must be careful not to eat too much starch.” (Health promoter)

Some participants explained that they have discussed oral health with patients and occasionally provided oral health counseling by both encouraging behavioral change and providing information about dental/oral health care.

“I will tell the client as we do bloods [phlebotomy] that it’s also important to look into the oral health. Just to prevent all these complications with the dental issues.” (Clinical nurse practitioner)

“I think all that gooey stuff that gets stuck in your teeth, I tell patients not to eat those things.” (Doctor)

Participants also understood potential barriers to oral health care facing their patients, including lack of available services, perceived lack of treatment options, and a lack of supplies necessary to address oral health needs.

“I have been told that at NY1 [clinic] there is only one dentist. So, I would assume that obviously affects the number of patients that can be seen on a day.” (Doctor)

“We have a limited supply of things that can help the mouth, so I mean, these days, we have no Nystatin drops or lozenges, so we have to give them fluconazole for candida, which is ridiculous. I mean, it doesn’t make sense to take a systemic drug for a local condition, and I think it has to do with supply issues, rather than a coding issue.” (Doctor)

#### Uncertainty in managing patients with oral health complaints

While participants did understand some factors contributing to poor oral health and barriers to dental care for the population that they serve, participants expressed uncertainty when providing care for patients with oral health complaints. This uncertainty involved their own knowledge and training, the referral process, services available to patients with referrals, and the follow up process. Many participants mentioned that while they did often address oral condition (*e*.*g*. dental abscesses, gingivitis, Kaposi sarcoma), their lack of training in oral health led to a lack of confidence in their approach. One participant discussed uncertainty in both diagnosis and treatment plan for a patient with a potential dental abscess.

“Usually I hope for the best [chuckles] because ideally, like it would be great if it was a dental abscess, they’re going to either pull the teeth or give them antibiotics, and you kind of like hope okay, I’m hoping the antibiotics makes a bit of a difference until at least they see them, or I’m hoping this is the right antibiotics that I am giving.” (Doctor)

Most participants described referring patients for oral health care, albeit with limited knowledge of the available services. The process ranged from providing the patient with a formal a letter to just explaining to the patient that they must seek oral health care. Participants generally were unfamiliar with the NYI dental clinic to which they were referring.

“I’ve been told by my colleagues that there is a dentist at NY1. I have got no idea where NY1 is, or what services are provided there, or what the waiting times for patients are. So, at the moment I’m not comfortable at all. All I can do is tell the patient, I mean, obviously if it’s not an emergency, I will say there is a dentist at NY1, you can go and seek assistance there.” (Doctor)

One participant stated that his or her lack of knowledge of available services causes him/her discomfort.

“I mean, it makes me a little bit uncomfortable that I don’t know exactly what services I am referring to, which isn’t great. I haven’t had much exposure to dentistry in the past. I’ve never had someone at the same facility who I can go and talk to, and like gain a bit of experience from. So when it comes to dentistry, I’m not very comfortable.” (Doctor)

Participants also stated that they do not have the ability to appropriately follow up with patients, so they are unsure whether those who they refer are receiving care.

### Perceptions of oral health interventions

#### Education

Despite the uncertainty in how to care for oral health, participants had multiple suggestions for how to deliver oral health education to providers. Participants presented strategies to gain more knowledge of oral health and dentistry including medical education reform, continuing education for medical professionals, and informal training provided to GCHC employees. Some participants also mentioned individual efforts to gain knowledge (e.g., reading textbooks about oral health). One participant suggested that educating providers on oral health prevention strategies might contribute to lowing costs, as well as improved health.

“It’s important for us to expand our knowledge on oral health because prevention is better than cure. In terms of costs, it’s a problem because if we are able to prevent the problem, then we are going to serve in terms of budget and stuff. For the patient as well, to save the patient’s time and money and all those things, to come to the clinic for the thing that was supposed to be prevented through health education.” (Clinical nurse practitioner)

Similar to the health counseling noted above, participants discussed the utility in providing oral health education to patients. Suggested interventions included direct communication between providers and patients, presentations on oral health topics by clinic staff, and provision of information for patients about where oral health services are available in their community.

“I think as doctors, we don’t address oral health, unless it’s a problem. Like especially with prevention, when we are talking about diet and stuff it’s probably a great idea to talk about brushing your teeth and if there are problems go to a dentist.” (Doctor)

Most participants felt strongly that health promoters are a valuable resource for providing health care information to patients. All participants stressed that health promoters should be trained to deliver oral health education as one of the medical topics

“We have health promoters, and we have a waiting area where there are lots of patients. I think that would also be a great area to actually talk to patients about things like oral health because first of all, the health promoters speak Xhosa, so what they say tends to hit home better than what I tell a patient.” (Doctor)

#### Expanded roles for providers

Some participants discussed integrating oral exams into routine patient care. One participant suggested that the oral exam should be included in the screening protocol.

“When we are triaging patients, we screen them for TB and things, so I think maybe screening for oral health would not be a bad idea. I mean, if we can screen for TB, why not?” (Doctor)

Another provider suggested that medical providers including oral exams into routine patient care could aid in preventing of oral disease.

“It could be education about oral hygiene and I guess a small examination of the teeth and the mouth can prevent a bigger problem. So then you can examine and if there is a problem you can defer to a dentist.” (Doctor)

While all participants were willing to expand their expertise to include oral health, some identified time constraints as a barrier to uptake. These participants stated that to meet their quota of about 30 patients per day, they were forced to spend far less time with each patient than they would like.

“All the doctors see 35, or 20 if you are an intern. So we don’t really have time to do more than just to get the complaint, treat, sort out, or refer the next patient. You have a very short time with every patient. But sometimes I educate them, but then to go beyond that, probably not.” (Doctor)

#### Colocation of services

Providers stressed the most effective intervention in increasing access to care would be establishing oral health services at GCHC. They suggested that lack of oral health services at the facility was a both a barrier to patients accessing oral health care and contributed to their own lack of oral health knowledge. Participants stated that colocation of care would increase efficiency in care delivery and improve patient experience. They explained how physical separation of medical and oral health services directly impacts patients in terms of limiting access to care.

“I mean just if everything is in one place, it’s easier to access. I mean, if a patient comes to see me for hypertension and they’ve got like a minor toothache, or minor dental complaint, I feel that they might get lost to follow-up if I say you have to go to NY1. I don’t know what the situation is there, go there and see what you can find out, whereas if I have a dentist at my facility, it will be like Dr So and So is the dentist, he or she is just over there, just pop around the corner and go and see him or her.” (Doctor)

Furthermore, participants discussed how colocation of medical and oral health care would improve efficiency and increase access to needed care.

“If the patient comes with a dental problem, that means we will refer the patient to the dentist, and it would be an advantage for the patient not to go back and forth. If there were a dentist at GCHC and the patient is coming for treatment like ARV, he or she could also be seen for the dental problem, in Gugulethu.” (Clinical Nurse Practitioner)

Participants also explained that colocation would allow for inter-provider communication. This direct communication between providers from different specialties allows for shared knowledge and case discussion. Participants stated that this communication is beneficial to them because they are then able to exchange information, discuss cases, and learn from one another.

“Having a dentist on side would help a lot, because it means I will know where to refer. It means if I have a question, I will know where to go and ask. It means if I’m struggling with something, like I’m not sure whether it’s an oral health problem or not then I will be able to go and ask that specific person that’s working there.”(Professional Nurse)

In addition to also improving patient experience, participants gave examples of other specialties, such as psychiatry, located at GCHC from which they benefited directly.

“If I need some advice from the psychiatrist who comes once a week, I can go when he’s here and say hi, can I have some help with something, and we can talk about whatever issue I have, or any of my staff have. But you can’t do it with the dentistry team because they’re not here.” (Doctor)

## Discussion

**Fig 1:**
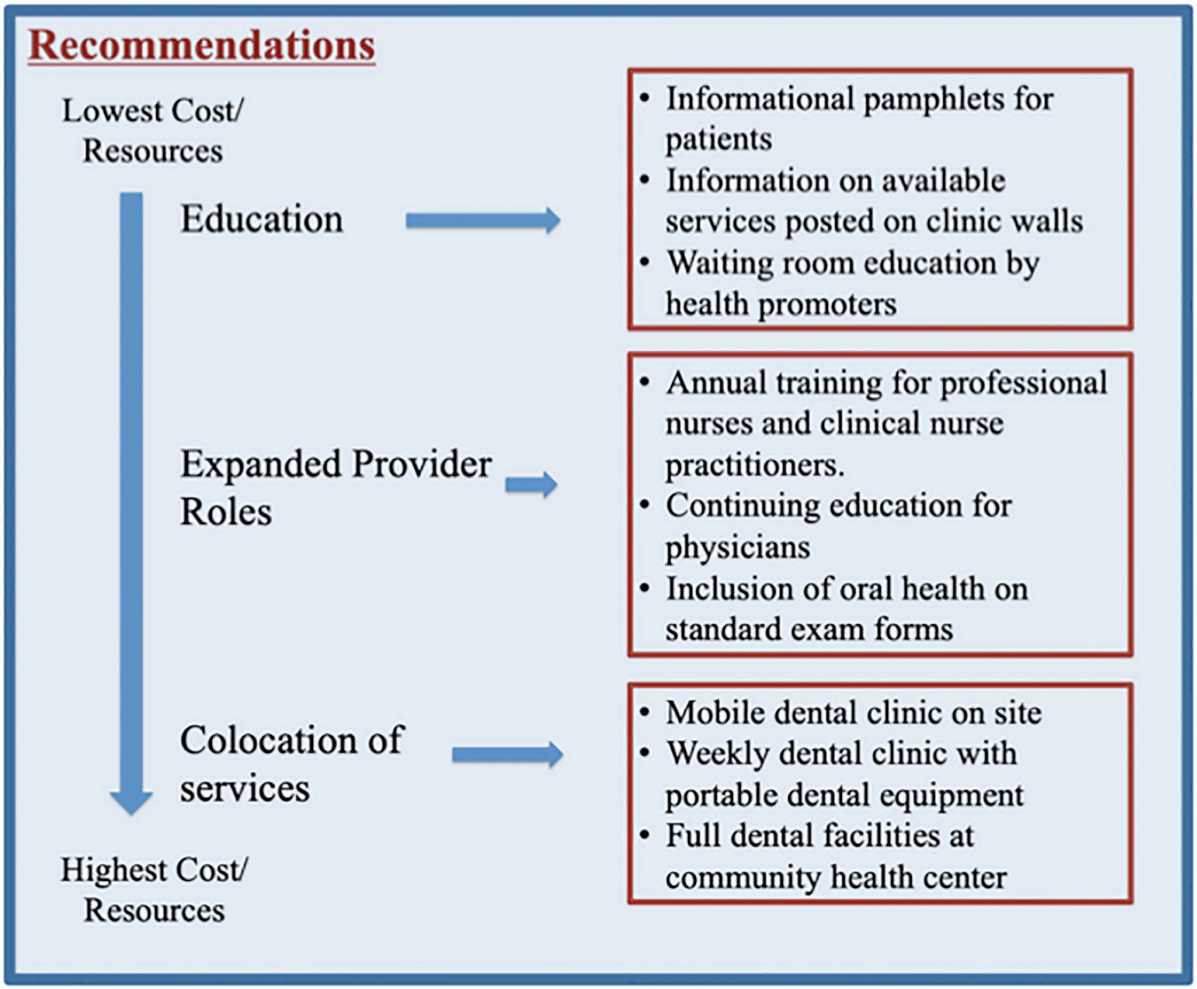
Proposed interventions categorized by level of required resources. These interventions are based on participants’ ideas not all of which were included in the representative quotes used in the manuscript.

In this qualitative study, we found that general medical providers at all levels of care in this particular setting—a community health center in a low-income area of South Africa—viewed oral health as an important aspect of overall health. Participants of this study recognized that they had insufficient knowledge to accurately manage, through triage, referral, or even treatment, patients with oral health complaints presenting to their clinics. Factors leading to this uncertainty ranged from lack of training, interprofessional communication, and time—a perceived barrier to care. Awareness and education are necessary for health professionals at all levels of care to triage and refer appropriately so that patients may access the oral health care they require. While participants suggested improving oral health required personal responsibility with respect to both hygiene and to some extent nutrition, they also recognized the barriers to resources necessary to achieve these goals associated with living in resource poor settings. Participants identified and discussed a wide variety of interventions aimed at increasing access and utilization of oral health care ranging from low to high cost, thus providing a framework for choosing reasonable interventions based on available resources (Fig 1). Interestingly, no differences were seen in themes across cadres of health professionals.

Our findings suggest that reliable and sustainable means to ensure appropriate oral health care delivery may include colocation of medical and oral health services and increased focus on prevention of oral disease at both medical and oral health facilities [16–18] Basic oral health counseling may also improve both oral and general health outcomes in communities lacking access to preventive oral health care or high quality oral health services. Streamlining care delivery and improving access to oral health care may improve patient care overall. In the United States, recognition of the need for improved access to quality dental services led to a push to increase integration of care. In 2011, the Dentaquest foundation set forth a continuum of integration models including the Early Childhood Oral Health program, which integrates oral health into primary care for the Medicaid population [19]. Other models involve expanding provider roles by training doctors to provide preventive oral health services such as placing fluoride varnish and preparing dentists to screen for systemic disease [20,21]. A 2018 systematic review suggests that several models of integrated care exist for pediatric populations in the United States, primarily in the setting of Federally Qualified Health Centers aiming to improve access to care for low-income communities [22]. A recent case study of four programs integrating preventive oral health services into primary care settings suggested that this activity serves to increase the delivery of oral health care and provides efficiency in care coordination [23].

Participants recognized the connection between oral and systemic health and may be able to easily target both oral health and noncommunicable diseases by addressing shared risk factors and building on clinic practices already in place. Oral disease and systemic, noncommunicable diseases (NCDs) such as hypertension and diabetes are linked, share common risk factors, and are prevalent among populations such as that served by participants of this study [12]. Furthermore, many systemic diseases (e.g. HIV) exhibit oral manifestations. Already, providers at GCHC counsel on behavior modification to prevent occurrence of NCDs and/or to improve the health status of those suffering from NCDs. Some even noted the link between systemic and oral disease. Coupled with education, providers may expand their routine primary care to include both a cursory oral exam and oral health counseling within standard behavior modification. While providers cited time as a barrier to expansion of provider roles, even solely recognizing shared risk factors between NCDs and oral disease would allow for more integrative behavior modification and nutritional counseling. Expanding and integrating care among both medical and dental providers may also help to alleviate the impact of provider shortage on patient access to care [20].

Education in basic oral health both during medical training and as continuing education was suggested as a possible intervention to better prepare providers to address and triage oral health issues [24,25]. While overarching institutional changes may be necessary to improve provider knowledge in the long-term, more immediate options include providing continuing education on common oral health issues to providers practicing in settings such as GCHC. Such programs can provide customized training on best practices for approaching oral health conditions that are common in each particular setting and population [25]. Training can also be tailored to all levels of provider ranging from the health promoter to nurses and doctors. GCHC, like many community health centers, provides one-day in service training courses for their employees on health topics relevant to their patient population. Inclusion of oral health training may lead to improved patient care based on increase provider knowledge.

This study has a number of strengths and limitations. It provides valuable information on a range of interventions to increase the quality and efficiency of oral health care available to residents of resource-poor communities relying on state sponsored medical services. The findings described here reflected an in-depth understanding of one clinic and included participants from all levels of medical care. Participants offered a wide range of potentially impactful interventions and reflected on their own knowledge, training, and patient care. Enthusiasm with respect to oral health care among the participants may have been influenced by the interview itself, thus limiting the findings presented here. Furthermore, findings reported here do not attempt to comment on prioritization relative to other potential initiatives within the clinic. The sample size was small; however, it was sufficient to reach thematic saturation. While qualitative studies are not generalizable due to the nature of the methodology, these results may be applicable to residents of similar communities across the region. Additional studies are needed from community-based samples in multiple settings to gain a deeper understanding of dental care knowledge and delivery among medical providers broadly. Next steps include harnessing this enthusiasm to develop and evaluate the efficacy of the suggested interventions to improve access to quality oral health care services.
