# Supplementary material for: Perceived oral health interventions by medical providers in Gugulethu, South Africa
Source: PLoS One. 2020 May 26;15(5):e0233437. doi: 10.1371/journal.pone.0233437 (PMC7250410; doi:10.1371/journal.pone.0233437)
Supplement: Attachment — Submitted filename: Response to Reviewers_Final.docx [file pone.0233437.s002.docx]

Response to Reviewers:

**Reviewer #1:**

*1. The Title may require revision to 'Perceived oral health interventions by medical providers in Gugulethu, South Africa'. It is not clear as it is*

**Response:** We thank the reviewer for the suggestion and have changed the title accordingly.

*2. Inductive based on which hypothesis or theory or premise?*

**Response:** We apologize for any confusion. We mean inductive based on grounded theory. The methods have been updated to state this explicitly (page 6):

“Factors influencing access to oral health care were identified using an inductive conventional content analytical approach based on grounded theory”

*3. Define a physician. In the South African context a physician is a trained specialist doctor with a masters degree. Rather the authors called it 'doctor'.*

**Response:** Thank you for this suggestion. We have replaced ‘physician’ with ‘doctor’ throughout the manuscript.

*4. Data found in the manuscript is only chosen excerpts. One hour long audiotapes and relevant transcripts were not accessed by Reviewer and are not in the manuscript.*

**Response:** Representative quotations are directly pulled from the transcripts were used and the authors are willing to provide the transcripts in their entirety should the editors require it.

*5. Local public sector health services are provided free-of-charge (Reference?)*

**Response:** The reviewer is correct. The following reference has been added to further describe the local healthcare system:

14. Earnshaw VA, Bogart LM, Courtney I, Zanoni H, Bangsberg DR, Orrell C, Goggin K, Katz IT. Exploring Treatment Needs and Expectations for People Living with HIV in South Africa: A Qualitative Study. AIDS Behav. 2018; doi:10.1007/s10461-018-2101-x.

*6. add 'with'. Experience with caring for ...*

**Response:** This change has been made.

*7. Data collection should be empirical in any research design. In qualitative designs, validity and robustness of data is substantiated by the following:
1. Trustworthiness
2. Credibility
3. Reflexivity and bracketing
I would suggest the authors add information on the above in the Methods section*.

Response: To address this comment the following was added to the methods section (page 5):

“Prior to any data collection, the interviewer (RFL) established rapport and trustworthiness at the clinic through several informal visits. His credibility was supported through an introduction by author CO, who has worked with the community for 20 years. To prevent reflexivity and bracketing, the RFL reassured staff that their opinions were highly valued and should not be influenced by himself or others.”

*8. I do not understand why is this sub-theme or category under the overall theme of ORAL HEALTH KNOWKEDGE. It seems to be related to PATIENT CARE , more so as it related to I access to care.*

**Response:** Thank you for this comment. This subtheme describes the knowledge the providers have of the social issues such as poverty that impact oral health. We therefore believe it belongs in the theme of Oral Health Knowledge. To clarify the meaning of this subtheme, we have changed the heading to “Understanding the impact of poverty on oral health.”

*9. Time constraints later become the authors' main point of discussion numerous times in the discussions. It is suggested that it noted as a stand-alone sub-theme under Patient Care as it is highlighted as the one of the main barriers.*

**Response:** In the discussion, we comment on how time constraints can impact 1) attaining sufficient knowledge (page 19) and 2) expanding provider roles (page 21). We appreciate the reviewer’s suggestion to make time constraints a stand-alone sub-theme; however, the participants spoke about time in the context of these other topics as currently presented in the Results. We therefore feel reorganization would not accurately capture their views.

*10. Time, a perceived barrier to care*

**Response:** This change has been made*.*

*11. Study Limitations?*

**Response:** Study limitations are listed in the last paragraph of the discussion. We have edited this section (page 22) in the following way to further clarify the limitations:

“This study has a number of strengths and limitations. It provides valuable information on a range of interventions to increase the quality and efficiency of oral health care available to residents of resource-poor communities relying on state sponsored medical services. The findings described here reflected an in-depth understanding of one clinic and included participants from all levels of medical care. Participants offered a wide range of potentially impactful interventions and reflected on their own knowledge, training, and patient care. Enthusiasm with respect to oral health care among the participants may have been influenced by the interview itself, thus limiting the findings presented here. Furthermore, findings reported here do not attempt to comment on prioritization relative to other potential initiatives within the clinic. Additional studies are needed from community-based samples in multiple settings to gain a deeper understanding of dental care knowledge and delivery among medical providers broadly. Next steps include harnessing this enthusiasm to develop and evaluate the efficacy of the suggested interventions to improve access to quality oral health care services.”

*12. Did gender or sex influence any of the perceptions as it is noted that there were more females than male participants.*

**Response:** The reviewer raises an interesting point; however, no participants mentioned gender, nor did we see patterns of perceptions by gender.

**Reviewer #2:**

*1. Introduction: First Paragraph – add oral health risk factors in the statement of “causal factors”*

**Response:** The sentence refers to the role that poverty, inequality, and systemic disease play in the epidemic of poor oral health. To clarify that point, we have edited the sentence in the first paragraph. It now reads (page 3):

“The World Health Organization (WHO) recognizes the need to address the epidemic of poor oral health, acknowledging poverty, inequality, and systemic disease as contributing factors, in addition to traditional oral health risk factors such as smoking and diet.”

*2. Introduction: Second Paragraph – Instead of using the phrase ‘developing countries’, I suggest using – low-income or high-income countries. Because all countries are developing, even high-income countries.*

**Response:** Thank you for this suggestion – the change has been made (page 3):

“Access to high quality oral health care is of particular concern in low-income countries and resource-poor communities due to limited infrastructure and a shortage trained oral health providers serving the public sector.”

*3. Methods: Study Site – What is a NYI Clinic?*

**Response:** NY1 Clinic is the name of the clinic where dental services are offered. This has been clarified in the following way (page 5):

“In Gugulethu, free oral health services are only provided at once facility called NY1 Clinic, which is affiliated with GCHC but located approximately one kilometer away.”

*4. Methods: Qualitative Data Collection – Why efforts were not made to conduct interviews in a local language? Do authors have full confidence that they did not miss information with English?*

**Response:** The participants all spoke English fluently, which we now clarify in the paper as follows (page 6):

*“*Interviews were conducted in a private location in English (as English is routinely used among health care professions and all participants were fluent in it)...

*5. It would have been helpful to get insights from the nearby dental clinic with regards to flow of patients, type of services provided and linkage between the two facilities.*

**Response:** We agree that insight from the dental clinic is valuable. Additional interviews were beyond the scope and resources of the current study; however, a future follow-up study will provide insight with respect to perceptions of interventions described here from the vantage point of oral health providers.
