# Supplementary material for: Perceived oral health interventions by medical providers in Gugulethu, South Africa
Source: PLoS One. 2020 May 26;15(5):e0233437. doi: 10.1371/journal.pone.0233437 (PMC7250410; doi:10.1371/journal.pone.0233437)
Supplement: Attachment — Submitted filename: Response to Reviewers2.docx [file pone.0233437.s003.docx]

Response to Review:

**Editor**: The PLOS Data policy requires authors to make all data underlying the findings described in their manuscript fully available without restriction, with rare exception (please refer to the Data Availability Statement in the manuscript PDF file). The data should be provided as part of the manuscript or its supporting information, or deposited to a public repository. For example, in addition to summary statistics, the data points behind means, medians and variance measures should be available. If there are restrictions on publicly sharing data—e.g. participant privacy or use of data from a third party—those must be specified.

**Response**: We appreciate the importance of sharing original data; however, we have concerns about participant anonymity (see Tsai et al, Soc Sci Med. 2016 Nov;169:191-198). We have therefore added the following to the Methods section of the manuscript (page 6):

“Full transcripts of the interviews and the codebook are available upon request and will be redacted appropriately to maintain anonymity of participants.”

**Reviewer #3:** *This is a well written article. The revisions to the manuscript are appropriate. I would like to request the authors to elaborate on the study limitations. Currently this section is very brief. Would there be other limitations of this work? can these findings be replicated to other settings? Are the study participants likely to reflect experiences of most patients in the clinic/region? These could be elaborated further.*

**Response:** We thank the reviewer for the suggestion and have added the following to the limitations section of the manuscript (page 22):

“The sample size was small; however, it was sufficient to reach thematic saturation. While qualitative studies are not generalizable due to the nature of the methodology, these results may be applicable to residents of similar communities across the region.”
