# Supplementary material for: Perceived oral health interventions by medical providers in Gugulethu, South Africa
Source: PLoS One. 2020 May 26;15(5):e0233437. doi: 10.1371/journal.pone.0233437 (PMC7250410; doi:10.1371/journal.pone.0233437)
Supplement: Peer review history

# Peer review: Decision Letter 0

**DOI:** 10.1371/journal.pone.0233437.r001

**Authors:** Janhavi Ajit Vaingankar

3 Jan 2020

PONE-D-19-29072

Perceptions of oral health interventions among medical providers in Gugulethu, South Africa

PLOS ONE

Dear Haberer,

Thank you for submitting your manuscript to PLOS ONE. After careful consideration, we feel that it has merit but does not fully meet PLOS ONE’s publication criteria as it currently stands. Therefore, we invite you to submit a revised version of the manuscript that addresses the points raised during the review process.

We would appreciate receiving your revised manuscript by Feb 16 2020 11:59PM. When you are ready to submit your revision, log on to https://www.editorialmanager.com/pone/ and select the 'Submissions Needing Revision' folder to locate your manuscript file.

If you would like to make changes to your financial disclosure, please include your updated statement in your cover letter.

To enhance the reproducibility of your results, we recommend that if applicable you deposit your laboratory protocols in protocols.io, where a protocol can be assigned its own identifier (DOI) such that it can be cited independently in the future. For instructions see: http://journals.plos.org/plosone/s/submission-guidelines#loc-laboratory-protocols

Please include the following items when submitting your revised manuscript:

- A rebuttal letter that responds to each point raised by the academic editor and reviewer(s). This letter should be uploaded as separate file and labeled 'Response to Reviewers'.
- A marked-up copy of your manuscript that highlights changes made to the original version. This file should be uploaded as separate file and labeled 'Revised Manuscript with Track Changes'.
- An unmarked version of your revised paper without tracked changes. This file should be uploaded as separate file and labeled 'Manuscript'.

Please note while forming your response, if your article is accepted, you may have the opportunity to make the peer review history publicly available. The record will include editor decision letters (with reviews) and your responses to reviewer comments. If eligible, we will contact you to opt in or out.

We look forward to receiving your revised manuscript.

Kind regards,

Janhavi Ajit Vaingankar

Academic Editor

PLOS ONE

Journal Requirements:

When submitting your revision, we need you to address these additional requirements:

1. Please ensure that your manuscript meets PLOS ONE's style requirements, including those for file naming. The PLOS ONE style templates can be found at http://www.plosone.org/attachments/PLOSOne_formatting_sample_main_body.pdf and http://www.plosone.org/attachments/PLOSOne_formatting_sample_title_authors_affiliations.pdf

2. Please include a copy of the topic/interview guide used in the study, in both the original language and English, as Supporting Information, or include a citation if it has been published previously.

Additional Editor Comments (if provided):

I would like to suggest that authors refer to COREQ guidelines and submit a COREQ checklist along with the revision. In particular, please elaborate on qualitative / academic skills of the research team members involved in data collection and analysis, reflexivity and data saturation. If possible, append the interview guide with the article. In addition, please discuss some limitations of this study.

[Note: HTML markup is below. Please do not edit.]

Reviewers' comments:

Reviewer's Responses to Questions

**Comments to the Author**

1. Is the manuscript technically sound, and do the data support the conclusions?

The manuscript must describe a technically sound piece of scientific research with data that supports the conclusions. Experiments must have been conducted rigorously, with appropriate controls, replication, and sample sizes. The conclusions must be drawn appropriately based on the data presented. 

Reviewer #1: Partly

Reviewer #2: Yes

**********

2. Has the statistical analysis been performed appropriately and rigorously? 

Reviewer #1: No

Reviewer #2: N/A

**********

3. Have the authors made all data underlying the findings in their manuscript fully available?

The PLOS Data policy requires authors to make all data underlying the findings described in their manuscript fully available without restriction, with rare exception (please refer to the Data Availability Statement in the manuscript PDF file). The data should be provided as part of the manuscript or its supporting information, or deposited to a public repository. For example, in addition to summary statistics, the data points behind means, medians and variance measures should be available. If there are restrictions on publicly sharing data—e.g. participant privacy or use of data from a third party—those must be specified.

Reviewer #1: No

Reviewer #2: No

**********

4. Is the manuscript presented in an intelligible fashion and written in standard English?

PLOS ONE does not copyedit accepted manuscripts, so the language in submitted articles must be clear, correct, and unambiguous. Any typographical or grammatical errors should be corrected at revision, so please note any specific errors here.

Reviewer #1: Yes

Reviewer #2: Yes

**********

5. Review Comments to the Author

Please use the space provided to explain your answers to the questions above. You may also include additional comments for the author, including concerns about dual publication, research ethics, or publication ethics. (Please upload your review as an attachment if it exceeds 20,000 characters)

Reviewer #1: Data collection should be empirical in any research design. In qualitative designs, validity and robustness of data is substantiated by the following:

1. Trustworthiness

2. Credibility

3. Reflexivity and bracketing

I would suggest the authors add information on the above in the the Methods section.

Reviewer #2: 1. Introduction: First Paragraph – add oral health risk factors in the statement of “causal factors”

2. Introduction: Second Paragraph – Instead of using the phrase ‘developing countries’, I suggest using – low-income or high-income countries. Because all countries are developing, even high-income countries.

3. Methods: Study Site – What is a NYI Clinic?

4. Methods: Qualitative Data Collection – Why efforts were not made to conduct interviews in a local language? Do authors have full confidence that they did not miss information with English?

5. It would have been helpful to get insights from the nearby dental clinic with regards to flow of patients, type of services provided and linkage between the two facilities.

**********

6. PLOS authors have the option to publish the peer review history of their article (what does this mean?). If published, this will include your full peer review and any attached files.

If you choose “no”, your identity will remain anonymous but your review may still be made public.

**Do you want your identity to be public for this peer review?** For information about this choice, including consent withdrawal, please see our Privacy Policy.

Reviewer #1: No

Reviewer #2: No

[NOTE: If reviewer comments were submitted as an attachment file, they will be attached to this email and accessible via the submission site. Please log into your account, locate the manuscript record, and check for the action link "View Attachments". If this link does not appear, there are no attachment files to be viewed.]

While revising your submission, please upload your figure files to the Preflight Analysis and Conversion Engine (PACE) digital diagnostic tool, https://pacev2.apexcovantage.com/. PACE helps ensure that figures meet PLOS requirements. To use PACE, you must first register as a user. Registration is free. Then, login and navigate to the UPLOAD tab, where you will find detailed instructions on how to use the tool. If you encounter any issues or have any questions when using PACE, please email us at figures@plos.org. Please note that Supporting Information files do not need this step.

Attachment Submitted filename: PONE-D-19-29072_reviewerME.pdf Click here for additional data file.

# Author comment: Author response to Decision Letter 0

**DOI:** 10.1371/journal.pone.0233437.r002

22 Jan 2020

Response to Reviewers:

Reviewer #1: 

1. The Title may require revision to 'Perceived oral health interventions by medical providers in Gugulethu, South Africa'. It is not clear as it is

Response: We thank the reviewer for the suggestion and have changed the title accordingly. 

2. Inductive based on which hypothesis or theory or premise?

Response: We apologize for any confusion. We mean inductive based on grounded theory. The methods have been updated to state this explicitly (page 6):

“Factors influencing access to oral health care were identified using an inductive conventional content analytical approach based on grounded theory”

3. Define a physician. In the South African context a physician is a trained specialist doctor with a masters degree. Rather the authors called it 'doctor'.

Response: Thank you for this suggestion. We have replaced ‘physician’ with ‘doctor’ throughout the manuscript. 

4. Data found in the manuscript is only chosen excerpts. One hour long audiotapes and relevant transcripts were not accessed by Reviewer and are not in the manuscript.

Response: Representative quotations are directly pulled from the transcripts were used and the authors are willing to provide the transcripts in their entirety should the editors require it. 

5. Local public sector health services are provided free-of-charge (Reference?)

Response: The reviewer is correct. The following reference has been added to further describe the local healthcare system: 

14. Earnshaw VA, Bogart LM, Courtney I, Zanoni H, Bangsberg DR, Orrell C, Goggin K, Katz IT. Exploring Treatment Needs and Expectations for People Living with HIV in South Africa: A Qualitative Study. AIDS Behav. 2018; doi:10.1007/s10461-018-2101-x.

6. add 'with'. Experience with caring for ...

Response: This change has been made.

7. Data collection should be empirical in any research design. In qualitative designs, validity and robustness of data is substantiated by the following:

1. Trustworthiness

2. Credibility

3. Reflexivity and bracketing

I would suggest the authors add information on the above in the Methods section.

Response: To address this comment the following was added to the methods section (page 5):

“Prior to any data collection, the interviewer (RFL) established rapport and trustworthiness at the clinic through several informal visits. His credibility was supported through an introduction by author CO, who has worked with the community for 20 years. To prevent reflexivity and bracketing, the RFL reassured staff that their opinions were highly valued and should not be influenced by himself or others.” 

8. I do not understand why is this sub-theme or category under the overall theme of ORAL HEALTH KNOWKEDGE. It seems to be related to PATIENT CARE , more so as it related to I access to care.

Response: Thank you for this comment. This subtheme describes the knowledge the providers have of the social issues such as poverty that impact oral health. We therefore believe it belongs in the theme of Oral Health Knowledge. To clarify the meaning of this subtheme, we have changed the heading to “Understanding the impact of poverty on oral health.” 

9. Time constraints later become the authors' main point of discussion numerous times in the discussions. It is suggested that it noted as a stand-alone sub-theme under Patient Care as it is highlighted as the one of the main barriers.

Response: In the discussion, we comment on how time constraints can impact 1) attaining sufficient knowledge (page 19) and 2) expanding provider roles (page 21). We appreciate the reviewer’s suggestion to make time constraints a stand-alone sub-theme; however, the participants spoke about time in the context of these other topics as currently presented in the Results. We therefore feel reorganization would not accurately capture their views. 

10. Time, a perceived barrier to care

Response: This change has been made. 

11. Study Limitations? 

Response: Study limitations are listed in the last paragraph of the discussion. We have edited this section (page 22) in the following way to further clarify the limitations:

“This study has a number of strengths and limitations. It provides valuable information on a range of interventions to increase the quality and efficiency of oral health care available to residents of resource-poor communities relying on state sponsored medical services. The findings described here reflected an in-depth understanding of one clinic and included participants from all levels of medical care. Participants offered a wide range of potentially impactful interventions and reflected on their own knowledge, training, and patient care. Enthusiasm with respect to oral health care among the participants may have been influenced by the interview itself, thus limiting the findings presented here. Furthermore, findings reported here do not attempt to comment on prioritization relative to other potential initiatives within the clinic. Additional studies are needed from community-based samples in multiple settings to gain a deeper understanding of dental care knowledge and delivery among medical providers broadly. Next steps include harnessing this enthusiasm to develop and evaluate the efficacy of the suggested interventions to improve access to quality oral health care services.”

12. Did gender or sex influence any of the perceptions as it is noted that there were more females than male participants.

Response: The reviewer raises an interesting point; however, no participants mentioned gender, nor did we see patterns of perceptions by gender.

Reviewer #2:

1. Introduction: First Paragraph – add oral health risk factors in the statement of “causal factors”

Response: The sentence refers to the role that poverty, inequality, and systemic disease play in the epidemic of poor oral health. To clarify that point, we have edited the sentence in the first paragraph. It now reads (page 3): 

“The World Health Organization (WHO) recognizes the need to address the epidemic of poor oral health, acknowledging poverty, inequality, and systemic disease as contributing factors, in addition to traditional oral health risk factors such as smoking and diet.”

2. Introduction: Second Paragraph – Instead of using the phrase ‘developing countries’, I suggest using – low-income or high-income countries. Because all countries are developing, even high-income countries.

Response: Thank you for this suggestion – the change has been made (page 3):

“Access to high quality oral health care is of particular concern in low-income countries and resource-poor communities due to limited infrastructure and a shortage trained oral health providers serving the public sector.”

3. Methods: Study Site – What is a NYI Clinic?

Response: NY1 Clinic is the name of the clinic where dental services are offered. This has been clarified in the following way (page 5): 

“In Gugulethu, free oral health services are only provided at once facility called NY1 Clinic, which is affiliated with GCHC but located approximately one kilometer away.”

4. Methods: Qualitative Data Collection – Why efforts were not made to conduct interviews in a local language? Do authors have full confidence that they did not miss information with English?

Response: The participants all spoke English fluently, which we now clarify in the paper as follows (page 6):

“Interviews were conducted in a private location in English (as English is routinely used among health care professions and all participants were fluent in it)... 

5. It would have been helpful to get insights from the nearby dental clinic with regards to flow of patients, type of services provided and linkage between the two facilities.

Response: We agree that insight from the dental clinic is valuable. Additional interviews were beyond the scope and resources of the current study; however, a future follow-up study will provide insight with respect to perceptions of interventions described here from the vantage point of oral health providers.

Attachment Submitted filename: Response to Reviewers_Final.docx Click here for additional data file.

# Peer review: Decision Letter 1

**DOI:** 10.1371/journal.pone.0233437.r003

**Authors:** Janhavi Ajit Vaingankar

14 Apr 2020

PONE-D-19-29072R1

Perceived oral health interventions among medical providers in Gugulethu, South Africa

PLOS ONE

Dear Haberer,

Thank you for submitting your manuscript to PLOS ONE. After careful consideration, we feel that it has merit but does not fully meet PLOS ONE’s publication criteria as it currently stands. Therefore, we invite you to submit a revised version of the manuscript that addresses the points raised during the review process.

We would appreciate receiving your revised manuscript by May 29 2020 11:59PM. When you are ready to submit your revision, log on to https://www.editorialmanager.com/pone/ and select the 'Submissions Needing Revision' folder to locate your manuscript file.

If you would like to make changes to your financial disclosure, please include your updated statement in your cover letter.

To enhance the reproducibility of your results, we recommend that if applicable you deposit your laboratory protocols in protocols.io, where a protocol can be assigned its own identifier (DOI) such that it can be cited independently in the future. For instructions see: http://journals.plos.org/plosone/s/submission-guidelines#loc-laboratory-protocols

Please include the following items when submitting your revised manuscript:

- A rebuttal letter that responds to each point raised by the academic editor and reviewer(s). This letter should be uploaded as separate file and labeled 'Response to Reviewers'.
- A marked-up copy of your manuscript that highlights changes made to the original version. This file should be uploaded as separate file and labeled 'Revised Manuscript with Track Changes'.
- An unmarked version of your revised paper without tracked changes. This file should be uploaded as separate file and labeled 'Manuscript'.

Please note while forming your response, if your article is accepted, you may have the opportunity to make the peer review history publicly available. The record will include editor decision letters (with reviews) and your responses to reviewer comments. If eligible, we will contact you to opt in or out.

We look forward to receiving your revised manuscript.

Kind regards,

Janhavi Ajit Vaingankar

Academic Editor

PLOS ONE

[Note: HTML markup is below. Please do not edit.]

Reviewers' comments:

Reviewer's Responses to Questions

**Comments to the Author**

1. If the authors have adequately addressed your comments raised in a previous round of review and you feel that this manuscript is now acceptable for publication, you may indicate that here to bypass the “Comments to the Author” section, enter your conflict of interest statement in the “Confidential to Editor” section, and submit your "Accept" recommendation.

Reviewer #2: All comments have been addressed

Reviewer #3: All comments have been addressed

**********

2. Is the manuscript technically sound, and do the data support the conclusions?

The manuscript must describe a technically sound piece of scientific research with data that supports the conclusions. Experiments must have been conducted rigorously, with appropriate controls, replication, and sample sizes. The conclusions must be drawn appropriately based on the data presented. 

Reviewer #2: Yes

Reviewer #3: Yes

**********

3. Has the statistical analysis been performed appropriately and rigorously? 

Reviewer #2: N/A

Reviewer #3: N/A

**********

4. Have the authors made all data underlying the findings in their manuscript fully available?

The PLOS Data policy requires authors to make all data underlying the findings described in their manuscript fully available without restriction, with rare exception (please refer to the Data Availability Statement in the manuscript PDF file). The data should be provided as part of the manuscript or its supporting information, or deposited to a public repository. For example, in addition to summary statistics, the data points behind means, medians and variance measures should be available. If there are restrictions on publicly sharing data—e.g. participant privacy or use of data from a third party—those must be specified.

Reviewer #2: No

Reviewer #3: No

**********

5. Is the manuscript presented in an intelligible fashion and written in standard English?

PLOS ONE does not copyedit accepted manuscripts, so the language in submitted articles must be clear, correct, and unambiguous. Any typographical or grammatical errors should be corrected at revision, so please note any specific errors here.

Reviewer #2: Yes

Reviewer #3: Yes

**********

6. Review Comments to the Author

Please use the space provided to explain your answers to the questions above. You may also include additional comments for the author, including concerns about dual publication, research ethics, or publication ethics. (Please upload your review as an attachment if it exceeds 20,000 characters)

Reviewer #2: (No Response)

Reviewer #3: This is a well written article. The revisions to the manuscript are appropriate. I would like to request the authors to elaborate on the study limitations. Currently this section is very brief. Would there be other limitations of this work? can these findings be replicated to other settings? Are the study participants likely to reflect experiences of most patients in the clinic/region? These could be elaborated further.

**********

7. PLOS authors have the option to publish the peer review history of their article (what does this mean?). If published, this will include your full peer review and any attached files.

If you choose “no”, your identity will remain anonymous but your review may still be made public.

**Do you want your identity to be public for this peer review?** For information about this choice, including consent withdrawal, please see our Privacy Policy.

Reviewer #2: No

Reviewer #3: No

[NOTE: If reviewer comments were submitted as an attachment file, they will be attached to this email and accessible via the submission site. Please log into your account, locate the manuscript record, and check for the action link "View Attachments". If this link does not appear, there are no attachment files to be viewed.]

While revising your submission, please upload your figure files to the Preflight Analysis and Conversion Engine (PACE) digital diagnostic tool, https://pacev2.apexcovantage.com/. PACE helps ensure that figures meet PLOS requirements. To use PACE, you must first register as a user. Registration is free. Then, login and navigate to the UPLOAD tab, where you will find detailed instructions on how to use the tool. If you encounter any issues or have any questions when using PACE, please email us at figures@plos.org. Please note that Supporting Information files do not need this step.

# Author comment: Author response to Decision Letter 1

**DOI:** 10.1371/journal.pone.0233437.r004

21 Apr 2020

Editor: The PLOS Data policy requires authors to make all data underlying the findings described in their manuscript fully available without restriction, with rare exception (please refer to the Data Availability Statement in the manuscript PDF file). The data should be provided as part of the manuscript or its supporting information, or deposited to a public repository. For example, in addition to summary statistics, the data points behind means, medians and variance measures should be available. If there are restrictions on publicly sharing data—e.g. participant privacy or use of data from a third party—those must be specified.

Response: We appreciate the importance of sharing original data; however, we have concerns about participant anonymity (see Tsai et al, Soc Sci Med. 2016 Nov;169:191-198). We have therefore added the following to the Methods section of the manuscript (page 6): 

“Full transcripts of the interviews and the codebook are available upon request and will be redacted appropriately to maintain anonymity of participants.”

Reviewer #3: This is a well written article. The revisions to the manuscript are appropriate. I would like to request the authors to elaborate on the study limitations. Currently this section is very brief. Would there be other limitations of this work? can these findings be replicated to other settings? Are the study participants likely to reflect experiences of most patients in the clinic/region? These could be elaborated further.

Response: We thank the reviewer for the suggestion and have added the following to the limitations section of the manuscript (page 22):

“The sample size was small; however, it was sufficient to reach thematic saturation. While qualitative studies are not generalizable due to the nature of the methodology, these results may be applicable to residents of similar communities across the region.”

Attachment Submitted filename: Response to Reviewers2.docx Click here for additional data file.

# Editor report: Decision Letter 2

**DOI:** 10.1371/journal.pone.0233437.r005

**Authors:** Janhavi Ajit Vaingankar

6 May 2020

Perceived oral health interventions by medical providers in Gugulethu, South Africa

PONE-D-19-29072R2

Dear Dr. Haberer,

We are pleased to inform you that your manuscript has been judged scientifically suitable for publication and will be formally accepted for publication once it complies with all outstanding technical requirements.

Within one week, you will receive an e-mail containing information on the amendments required prior to publication. When all required modifications have been addressed, you will receive a formal acceptance letter and your manuscript will proceed to our production department and be scheduled for publication.

Shortly after the formal acceptance letter is sent, an invoice for payment will follow. To ensure an efficient production and billing process, please log into Editorial Manager at https://www.editorialmanager.com/pone/, click the "Update My Information" link at the top of the page, and update your user information. If you have any billing related questions, please contact our Author Billing department directly at authorbilling@plos.org.

If your institution or institutions have a press office, please notify them about your upcoming paper to enable them to help maximize its impact. If they will be preparing press materials for this manuscript, you must inform our press team as soon as possible and no later than 48 hours after receiving the formal acceptance. Your manuscript will remain under strict press embargo until 2 pm Eastern Time on the date of publication. For more information, please contact onepress@plos.org.

With kind regards,

Janhavi Ajit Vaingankar

Academic Editor

PLOS ONE

# Editor report: Acceptance letter

**DOI:** 10.1371/journal.pone.0233437.r006

**Authors:** Janhavi Ajit Vaingankar

14 May 2020

PONE-D-19-29072R2 

Perceived oral health interventions by medical providers in Gugulethu, South Africa 

Dear Dr. Haberer:

I am pleased to inform you that your manuscript has been deemed suitable for publication in PLOS ONE. Congratulations! Your manuscript is now with our production department. 

If your institution or institutions have a press office, please notify them about your upcoming paper at this point, to enable them to help maximize its impact. If they will be preparing press materials for this manuscript, please inform our press team within the next 48 hours. Your manuscript will remain under strict press embargo until 2 pm Eastern Time on the date of publication. For more information please contact onepress@plos.org.

For any other questions or concerns, please email plosone@plos.org. 

Thank you for submitting your work to PLOS ONE.

With kind regards,

PLOS ONE Editorial Office Staff

on behalf of

Ms Janhavi Ajit Vaingankar 

Academic Editor

PLOS ONE
